# A mathematical model of combined CD8 T cell costimulation by 4-1BB (CD137) and OX40 (CD134) receptors

**DOI:** 10.1038/s41598-019-47333-y

**Published:** 2019-07-26

**Authors:** Anna Konstorum, Anthony T. Vella, Adam J. Adler, Reinhard C. Laubenbacher

**Affiliations:** 1 Center for Quantitative Medicine, School of Medicine, UConn Health, 263 Farmington Ave., Farmington, CT USA; 2 Department of Immunology, School of Medicine, UConn Health, 263 Farmington Ave., Farmington, CT USA; 3 Jackson Laboratory for Genomic Medicine, 263 Farmington Ave., Farmington, CT USA

**Keywords:** Cancer models, Immunization, Applied mathematics

## Abstract

Combined agonist stimulation of the TNFR costimulatory receptors 4-1BB (CD137) and OX40(CD134) has been shown to generate supereffector CD8 T cells that clonally expand to greater levels, survive longer, and produce a greater quantity of cytokines compared to T cells stimulated with an agonist of either costimulatory receptor individually. In order to understand the mechanisms for this effect, we have created a mathematical model for the activation of the CD8 T cell intracellular signaling network by mono- or dual-costimulation. We show that supereffector status is generated via downstream interacting pathways that are activated upon engagement of both receptors, and *in silico* simulations of the model are supported by published experimental results. The model can thus be used to identify critical molecular targets of T cell dual-costimulation in the context of cancer immunotherapy.

## Introduction

It is now well-understood that T lymphocytes respond not only to antigens derived from infectious agents, but also to (tumor-specific and tumor-associated) antigens^1^. An improper response by T cells can result in infection or tumor growth (if T cells are not properly activated) or autoimmune disease (if the T cells treat healthy host cells as infected)^2,3^.

T cell receptors are transmembrane receptors that play a critical role in modifying the T cell response after innate immune cells have presented antigen to the T Cell Receptor (TCR). Activation of CD28, the most well-known costimulatory receptor^4^, results in T cell proliferation, cytokine production, and other pro-effector phenotypic traits. Other costimulatory receptors include OX40 (CD134), 4-1BB (CD137), and CD27^5^. T cells also express checkpoint inhibitory receptors, which are necessary to prevent auto-immune disorders, the most well-studied of which are CTLA-4 and PD-1 due to their over-activation by certain tumors^6^. These receptors inhibit overlapping pathways, not all of which have been identified experimentally^7^. Predicting the phenotypic outcome of combinatorial costimulatory receptor activation is not currently possible but highly desired, especially in the context of drug development for cancer immunotherapy. For example, pharmacological agonist antibodies against OX40 and 4-1BB T cell costimulatory receptors have been found to be effective individually^8,9^ in improving systemic immune response and reducing tumor burden. Additionally, their effect when given in combination (termed dual costimulation) is cooperative in mitigating tumor growth^10–12^ and shows synergism with respect to CD8 T cell effector status^13,14^. Indeed, a combination therapy targeting both receptors in the context of several cancers is currently in clinical trials^15^. Nevertheless, the molecular mechanisms by which the effect of dual costimulation occurs have not been completely elucidated. Both 4-1BB and OX40 costimulatory receptors belong to the Tumor Necrosis Factor Receptor (TNFR) family, and act by binding to TNF receptor-associated factor (TRAF)-acting proteins, but they bind a different subset of TRAFs^16^, signal through overlapping^5,16^, and non-overlapping^17^ pathways, and preferentially activate different subsets of lymphocytes^5^. Therefore, their dual action in generating supereffector T cells may occur at either the intracellular, network-level scale via optimized network activation, at the population-scale via activation of the major subsets of T cells, or both.

Cancer immunotherapy refers to development of drugs to stimulate the immune system to fight the tumor and is a highly active area in basic and clinical cancer therapeutics research^18^. Since the effect of combining various immunotherapy drugs is difficult to predict, mathematical models of T-cell modulation by costimulatory receptor activity can help scientific researchers to build a more complete understanding of this process, and thereby design optimal combination therapies of immunotherapy drugs^19^.

While there has been a strong modeling effort to determine TCR specificity with respect to antigen binding (e.g.^20–22^ and see^23^ for review), modeling of downstream signaling pathways of the TCR and costimulatory receptors has been more sparse. Saez-Rodriguez *et al*.^24^ developed and partially validated a comprehensive Boolean network model of TCR and CD28 activation in CD4 and CD8 T cells, which was later extended by Beyer *et al*.^25^ to include an IL-2 receptor signaling module. Saadatpour *et al*.^26^ also developed a dynamic Boolean model for CD8 signaling in the context of T cell large granular lymphocyte (T-LGL) leukemia. The models all provided insight into dynamics of specific downstream pathways in T cell signaling, but did not include the TNFR family receptors 4-1BB or OX40 or their downstream components.

## Results

### Model for intracellular activation of CD8 T cells by OX40 and 4-1BB receptor agonists

**Figure 1:**
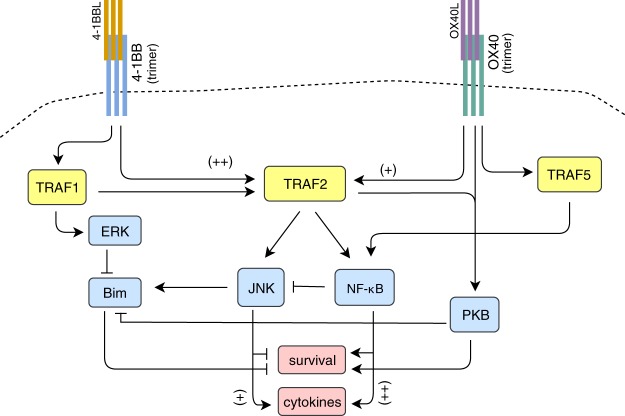
A model for T cell costimulation by the agonists of 4-1BB and OX40. TRAF proteins, which are activated by 4-1BB and OX40, are labeled in yellow, their downstream effectors, Bim, JNK, and NF-*κ*B are in blue, and the phenotypic outcomes: cellular survival and cytokine production, are in pink. Stimulation of T cells via their TCR receptor is assumed for the course of the simulation. Shown is stimulation of 4-1BB and OX40 via their natural ligands, activation via antibodies can also take place.

We present a model of intracellular CD8 T cell activation by OX40 and 4-1BB agonists using a stochastic multistate discrete logic framework to represent key molecules involved in the downstream signaling pathways of OX40 and/or 4-1BB, as summarized in Fig. 1. This framework allows for the representation of qualitative relationships between variables and requires much fewer parameters and kinetic information to develop than continuous models. Logic-based models have been used to investigate and elucidate a variety of signaling networks^27,28^. In such a system, state transitions can be represented using transition tables, where all possible combinations of input values for contributing variables are assigned an output state for a given species. A probability update function is assigned for each update rule (which is specified by a transition table) in order to more realistically represent the biological scenario where not all possible interactions may occur at each time step. Derivation of the transition tables for the modeled species, as well as the simulation methods, are described in the Methods. A more detailed discussion of the mechanics of the discrete model can be found in the Supplementary Materials.

Since the data that we use for development of the model comes from a variety of literature sources, many of which are qualitative in nature, we make a number of simplifying assumptions in order to reduce the parameter burden. First, we focus our goal with respect to the model such that it can be used to explain the synergy of 4-1BB and OX40 activation. The model components were chosen based on primary literature in the field of downstream signaling for 4-1BB and/or OX40 that contained appropriate information to build the dynamic interactions for the model. Development of the transition tables was based on literature-derived specific pathway interactions (such as ligation of OX40 that leads to subsequent activation to TRAF5^29^ or inhibition of survival by Bim^30^). Outcomes of mono- or dual-costimulation on survival, cytokine release, or activation of any particular molecular species were determined by the cumulative effect of multiple interacting pathway components, which were simulated by the model. References utilized during the model development phase are distinct from validation references.

Further, we assume optimal TCR activation, as many studies that we use to develop and validate our model use a vaccine strategy to ensure a large bolus of TCR triggering. Indeed, TCR activation is required for 4-1BB and OX40 upregulation, and thus is assumed to occur in all simulations of the model. We also consider that while knocked-down or overexpressed signaling components will continue to fluctuate in *in vitro* or *in vivo* settings, these fluctuations will be of an order lower than under normal conditions, hence due to the coarse-grained nature of our model, we take perturbed values of these signaling components to be constant (‘high’ or’low’, for overexpression or knock-out, respectively) during the course of the simulation. Further, we note that while OX40 is considered to be more active in CD4 (T helper) cells, and 4-1BB in CD8 (cytotoxic) T cells^31^, OX40 activity has been found to be important for activated CD8 T cell survival, proliferation, and cytokine production (even independently of CD4 T cell involvement)^32,33^, therefore we model the action of both receptors on CD8 T cells. Additionally, expression of OX40 and 4-1BB peaks at approximately 48 hours after antigen presentation to naive T cells^5^. Coexpression of the receptors is likely to continue for approximately 2–4 days post-activation^34,35^ which represents a critical phase of effector T cell differentiation, and is thus clinically important for development of sufficient numbers of these antigen-specific cytotoxic T cells^36^. Thus, focusing the modeling effort on this time period represents an efficient effort to understand and simulate a critical phase of T cell differentiation during which OX40 and 4-1BB play an important role in the quality of T cell effector generation. We take the expression and activation of the coreceptors to be constant during this peak period. More generally, our model may be considered to represent the relevant time period during which therapeutic response is elicited from mono- or dual-costimulation, and this period may be extended if the therapy is administered multiple times over longer periods^12,37^. Finally, OX40 and 4-1BB can be activated endogenously by their respective ligands OX40L and 4-1BBL, which are expressed on activated APCs such as DCs, B cells, and macrophages, or exogenously by monoclonal agonist antibodies^38^. While our model does not distinguish endogenous or exogenous activation of the receptors, it can serve as a framework for developing the distinction if desired. We assume baseline production and decay rates of approximately the same order for the variables, hence we do not include either in the model.

### Dual costimulation in the baseline model

**Figure 2:**
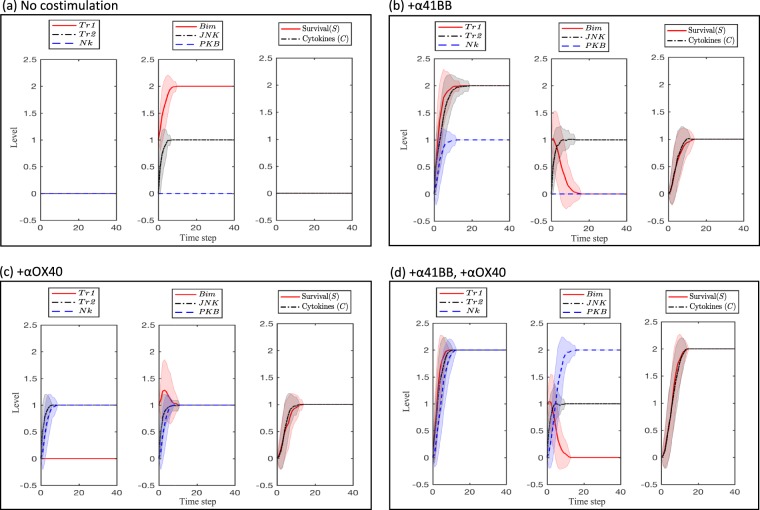
Behavior of each species during conditions of (**a**) no costimulation, (**b**) 4-1BB mono-costimulation, (**c**) OX40 mono-costimulation, and (**d**) dual costimulation. Low, medium, and high activity of molecular species are represented by Levels 0, 1, and 2, respectively. The solid line and shaded region represent, respectively, the mean and standard deviation of 100 simulations.

By varying 4-1BB (*I*) and OX40 (*Ox*) we consider behavior of the model under conditions of no costimulation (*I* = *Ox* = low), mono-costimulation (*I* = low and *Ox* = high or *I* = high and *Ox* = low), and dual-costimulation (*I* = *Ox* = high) (Fig. 2). We observe that under conditions of no costimulation, the TRAF proteins are not activated, and hence NF-*κ*B (*Nk*) is at its lowest level, and *Bim* at its highest. *JNK* maintains an intermediate level since, while it is not being activated by TRAF2 (*Tr*2), it is also not being inhibited by *Nk*. *Bim* is at a low level due to lack of *Ox*. Survival (*S*), since it is an outcome of the balance between activators *Nk* and *PKB* and inhibitors *Bim* and *JNK*, is at its lowest level (Fig. 2a). Cytokine release (*C*) is similarly low due to the absence of *Nk* (a moderate level of *JNK* with a low level of *Nk* is not sufficient to raise *C* levels to moderate).

Upon mono-costimulation with *I* (Fig. 2b), *S* increases to a moderate level due to a moderate increase in *Nk* (an outcome of higher *Tr*2 and a *I*-mediated decrease in *Bim*). Cytokine production shows a similarly modest increase due to the increase in *Nk*. The model thus predicts that upon mono-costimulation with one agonist, CD8 T cells will increase cytokine production and survival to a moderate extent. These results are consistent with experimentally observed results of increased survival and cytokine production in CD8 T cells from healthy donors^39^ and melanoma tumor-infilatrating CD8 lymphocytes^40^ when exposed to 4-1BB costimulatory ligand or agonist. Survival also increases to a moderate level upon mono-costimulation with *Ox* (Fig. 2c), this time due to an increase in *PKB*, alongside with an increase in *Nk*, and a moderate decrease in *Bim*. Cytokine production is increased to a moderate level due to the increase in *Nk*. Increased CD8 T cell survival and cytokine production have been been found to be increased upon costimulation by an OX40 agonist in an adoptive-trasferred OT-I CD8 T cell model^32,41^.

Dual costimulation results in the highest survival level due to high *NK* and *PKB* activity, and low *Bim* activity (Fig. 2d). Therefore, the model predicts that multiple pathways converge to maximize survival in the dual costimulation system. The strong increase in survival and cytokine production in dual-costimulated cells over mono-costimulated cells was observed in Lee *et al*.^13^ using an OT-I T cell transfer model. The group found that mono- and dual-costimulated CD8 T cells were accumulating to different extents - with greater accumulation by dual-costimulated cells (Fig. 4b^13^). Thus, the group found that the more profound CD8 T cell clonal expansion elicited by dual- compared to mono-costimulation during *in vivo* immunization (Fig. 4b^13^) could not be explained by enhanced proliferation. More specifically, when two complimentary flow cytometry-based methods were used to measure CD8 T cell proliferation (dilution of the fluorescent tracking dye CFSE, and incorporation of the deoxynucleotide analogue BrdU into replicating DNA), no differences were observed between dual- and mono-costimulation treatments at either the early, middle or late phases of the proliferative response (spanning from 0 to 105 hours, Fig. 4a,c,d^13^). Taken together, these results indicated that the effect of dual-costimulation in augmenting CD8 T cell clonal expansion, compared to either mono-costimulation, was due to its ability to program enhanced survival. Moreover, the group found that neither clonal expansion nor cytokine production was dependent on CD4 help (Fig. 6^13^), highlighting the criticality of intracellular cascades mediated by OX40 and 4-1BB in CD8 T cell supereffector generation.

### Effect of knockout on the effectiveness of dual-costimulation

**Figure 3:**
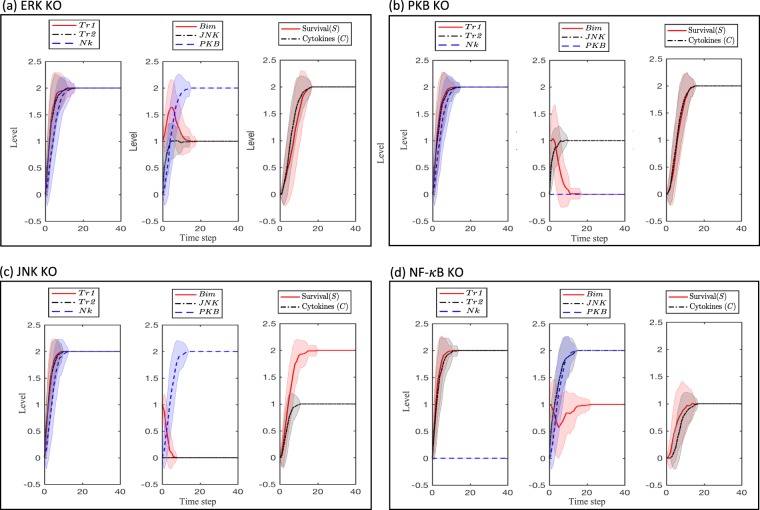
Simulating knock-out experiments of (**a**) *ERK*, (**b**) *PKB*, (**c**) *JNK*, and (**d**) *Nk* for dual-costimulated cells. The solid line and shaded region represent, respectively, the mean and standard deviation of 100 simulations.

Lee *et al*.^42^ cultured OT-I CD8 T cells that had been adoptively transferred into C57BL/6 mice, immunized with antigen and dual-costimulated with OX40 and 4-1BB, and subsequently purified from spleen, with a number of inhibitors to signaling cascades including PD98059 and U0126 (inhibitors of upstream regulators of ERK), Wortmannin and LY294002 (inhibitors of PI-3K, which is necessary for PKB stimulation), SP500125 (a JNK inhibitor), and Bay11 (an NF-*κ*B inhibitor). The group found that only Bay11 blocked the dual-costimulation-induced cell survival of the OT-I CD8 T cells. We thus decided to simulate this set of experiments by knocking-out *ERK*, *PKB*, *JNK*, or *Nk* (Fig. 3). The model predicts that only *Nk* knock-out results in a decrease in cell survival, similar to the observations by Lee *et al*.^42^. Our model can help to explain why only the NF-*κ*B inhibitor is capable of reducing dual-costimulated T cell survival: the remainder of the species can compensate for *ERK*, *PKB*, or *JNK* knockouts in dual-costimulated cells so that survival is not impacted, but *Nk* knock-out not only removes *Nk* from promoting survival, but also from acting on *JNK* (and thereby *Bim*) to minimize their antaganostic impact on survival. Only *PKB* is not affected, but it cannot completely compensate for the loss of *Nk*, and gain in *JNK* and *Bim*.

### Cellular phenotype as predictor of response to mono- and dual-costimulation

**Figure 4:**
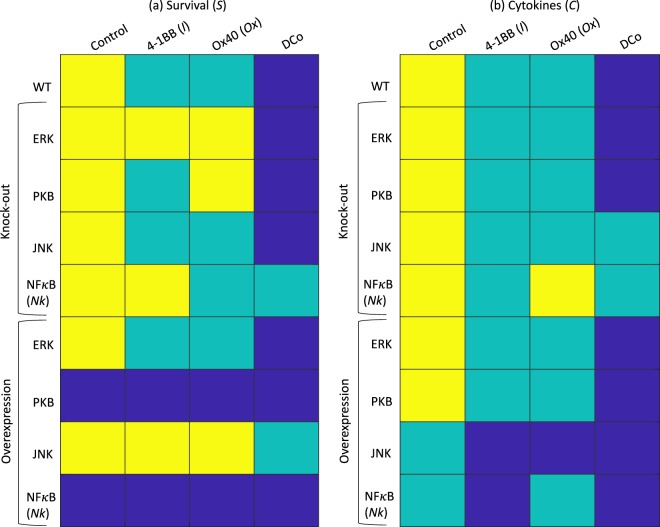
Steady-state (**a**) Survival (*S*) and (**b**) Cytokine release (*C*) in simulated cells that have been costimulated with 4-1BB (*I*) and/or OX40 (*Ox*) under knock-out or overexpression conditions of *ERK*, *PKB*, *JNK*, or *Nk*. If the model species name differs from the biological name, the model species name is in parentheses.

A major challenge in cancer immunotherapy (and, cancer therapy in general) is predicting the responsiveness of the patient to a particular immunotherapeutic treatment^43,44^. With the advent of tumor single-cell sequencing methods^45,46^, it may be possible to predict responsiveness to therapy based on the transcriptomic profiles of tumor and tumor-resident cells, a fraction of which are CD8 T cells. We consider this challenge by asking whether a CD8 T cell with perturbation in one of the four major downstream pathways of 4-1BB (*I*) and/or OX40 (*Ox*) (*ERK*, *JNK*, *PKB*, or *NK*) would be more or less responsive to mono- or dual-costimulation treatment (Fig. 4). The subsequent results represent predictions beyond what is currently studied in the experimental literature, but provide testable predictions for future experimental investigation.

While the model predicts that only *Nk* knock-out will abrogate the dual-costimulation response, further predictions include that *ERK* knock-out and *JNK* overexpression will abrogate survival mono-costimulation responses to both agonists, and *PKB* knock-out will abrogate the survival response to *Ox* while *Nk* knockout will abrogate survival response to *I* and cytokine response to *Ox*. Interestingly, *PKB* overexpression is predicted to result in maximal survival independent of treatment, but cytokine response will still depend on treatment, whereas *JNK* overexpression will lower survival responsiveness while increasing cytokine expression in response to stimulation in comparison to wild-type cells. Unsurprisingly, *Nk* overexpression is predicted to yield the highest survival and cyotokine expression independent of treatment in comparison to all other knock-out or overexpression experiments. The model has thus generated predictions with respect to responsiveness of T cells to mono- or dual-costimulation given knowledge of the activity of these pathways. For example, in cells with inhibition of the *ERK* pathway, mono- and dual-costimulation are predicted to increase cytokine expression, but only dual-costimulation would significantly impact survival. On the other hand, cells with overactive *PKB* can be expected to survive longer independent of treatment, but be responsive to treatment with respect to cytokine secretion. These predictions can help to guide experimental investigation towards a more comprehensive understanding of the influence of CD8 T cell phenotype to treatment response.

## Discussion

In this work, we have developed a stochastic multistate discrete model of CD8 T cell costimulatory receptor dual costimulation by agonists 4-1BB and OX40. Using *in silico* simulations of agonist stimulation, we have shown that the model can capture the experimental results observed for both mono- and dual-costimulation. We have further shown that the model can identify the NF-*κ*B pathway as the strongest contributor to the dual costimulation effect, which was shown experimentally by Lee *et al*.^42^. Finally, we have simulated response of the model to knock-out or overexpression conditions for key pathways in order to generate predictions of the responsiveness of T cells with heterogeneous phenotypes to both mono- and dual-costimulation.

We note that while there several methods to quantify synergy^47^, none of them account for discrete models. While we are unable to quantify the extent of synergy that may occur with dual costimulation over mono costimulation, we do observe that the model replicates the qualitative increase in response when the two drugs are given in combination in comparison to just one drug. Moreover, we surmise that superadditivity is present in the response, at least in survival, based on the following argument: we observe that in *ERK* knock-out conditions (which boost *BIM* activity), there is no longer any increase in survival in mono costimulation conditions, and a maximal increase in survival in dual costimulation conditions (Fig. 4(a)), which strongly suggests synergy (by any measure of it), and shows that dual costimulation allows the system to overcome the knock-out-induced increase in *BIM*-mediated pro-apoptotic activity. Therefore, we can conclude that even in wild-type conditions, where *ERK* activity may be increased depending on the costimulation provided, and therefore survival may be boosted further by the consequent lowering of *BIM* activity, there is already synergy in survival response to dual costimulation even in presence of *BIM* activity. While we do not see similar results with the cytokine expression, we can still conclude that there is a maximization of this response in dual costimulation vs. mono costimulation - and the extent to which it occurs (superadditive or additive) will require a more fine-grained model to assess.

We focus on the survival and cytokine pathways for CD8 T cell costimulation, because the available data is the most clear on these two phenotypic outcomes for both mono- and dual-costimulation of these cells. For example, Lee *et al*.^13^ observed that increase in dual costimulation-mediated T cell number was not due to cell cycle entry, indicating that the survival pathways are more critical to the observed changes in dual costimulation-mediated cell number than an increase in proliferation. Importantly, the model can not only capture the strong response of CD8 T cells to dual costimulation that was observed by Lee *et al*.^13^, but it can also be used to understand the molecular underpinnings of the supereffector response.

Of the central pathways downstream of the TRAF proteins involved in the dual costimulation response, NF-*κ*B was found to be a critical player (Fig. 3). It is conceivable that NF-*κ*B may play a more central role in immunotherapy synergy if a broader range of targets is considered. Indeed, the NF-*κ*B pathway has been found to be critical for T-cell elimination of tumors *in vivo*^48^, and CTLA-4 has been shown to inhibit TCR-mediated NF-*κ*B^49^. It thus may be of interest to understand and model how to optimize NF-*κ*B stimulation via a combination of agonists (e.g. OX40 and 4-1BB) and antagonists (e.g. CTLA-4 and PD-1), and how the responsivity of the T cells to these therapies may change under conditions of tumor-mediated immunoediting, as tumors are known to secrete factors that may inhibit TCR-mediated NF-*κ*B activation (and, potentially, OX40- and 4-1BB-mediated NF-*κ*B activation). More generally, incorporation of CTLA-4 and PD-1 pathways into the model may be of interested for future work in order to better understand whether the activation of these checkpoint inhibitory pathways in T cells may interfere with respect to mono- or dual-costimulation effectiveness of OX40 and/or 4-1BB agonists.

There were a number of biological details that we chose to omit from the model, but may be included in the future. For example, our assumption of optimal TCR triggering is based on the experimental studies that we have used to build our model. A more graded response to mono- and dual-costimulation under conditions of sub-optimal triggering would be of value to model with appropriate experimental evidence, since this is likely what occurs in an *in vivo* setting. Similarly, graded mono- and dual-costimulation (in comparison to maximal, as we model) may be also considered for future modeling efforts with appropriate experimental validation, since it is of interest to consider how dosage of one or both agonists impacts the costimulation response. Further, it has been shown that NF-*κ*B is a transcription factor for TRAF1 and TRAF2^50,51^. We chose not to include this feedback since its strength and time-course have not been evaluated in T cells, thus we do not have information on what role it plays in this system. Similarly, increase in cytokine production can theoretically result in autocrine feedback loops (e.g. binding of TNF*α* to the TNFR) that could further boost activation of the molecular actors in our model, since TNFR signals through TRAF2 (such an autocrine feedback loop was recently shown to exist for Treg cells^52^). Since we do not have information about the activity of such feedback loops for our no-costimulation model, and if such loops would be promoted further upon mono- or dual costimulation, we do not include this hypothetical feedback loop in our model. Nevertheless, even without incorporation of these factors, we still observe a maximal intracellular response to dual costimulation in the model, indicating that these factors may not be critical for the experimentally observed dual costimulation effect.

The CD8 T cell model of dual costimulation signaling may be relevant to therapeutic scenarios such as CAR T cell therapy where chimeric antigen receptors can be engineered to contain signaling motifs derived from different costimulatory receptors^53^. In this case, understanding of the mechanisms by which dual costimulation optimally boosts T cell response can be used to better understand how, and under what intracellular conditions, a CAR designed with OX40 and 4-1BB signaling motifs would optimally promote expansion of supereffector T cells. Moreover, OX40 and 4-1BB are expressed on activated CD4 (T helper) and constitutively on FoxP3^+^ CD25^+^ CD4^+^ (Treg) cells^54–57^. The intercellular interactions between these and CD8 cells in a dual costimulation environment will impact CD8 activity, as well as more broadly alter the immunogeneicity of tumors in which these cells act. Therefore, development of a multi-scale model encompassing both intracellular signaling cascades activated by dual costimulation and intercellular interactions potentiated in this environment will generate a mechanistic framework from which to disentangle the effect of the intra- vs. inter-cellular interactions on dual costimulation-mediated T cell costimulation, as well as to simulate conditions under which the population will be most (or, least) responsive to dual costimulation in order to create predictive models of dual costimulation efficacy. The intracellular dual costimulation CD8 T cell model presented here provides a first step in this direction.

## Methods

The model was simulated in Matlab R2017A using an asynchronous stochastic update scheme. Initial conditions were set to baseline physiological levels before 4-1BB (*I*) and/or OX40 (*Ox*)-mediated changes in molecular species activity. Notably, initial conditions (aside from *I* and *Ox*) did not impact the steady states. We provide an overview of the modeling scheme here, and provide more detailed modeling and simulation methods, including methods for the modification of the model for knock-out and overexpression experiments, in the Supplementary Material.

**Figure 5:**
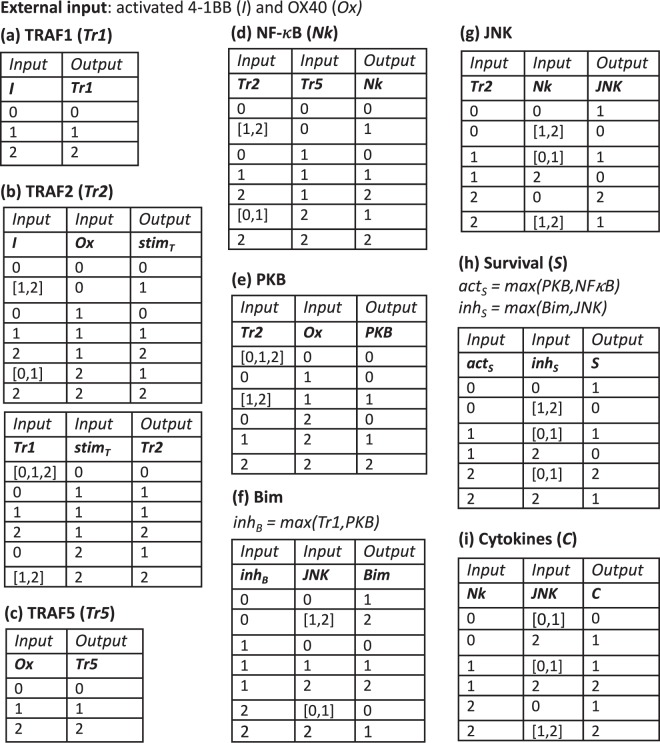
Multistate rule specification for the model of dual costimulation of CD8 T cells. Transition tables are displayed for each modeled species.

System variables, which include the downstream molecular species of 4-1BB and OX40, can take on three levels of activity: low, medium, or high. These levels are represented with the values of 0, 1, and 2, respectively. A state vector at time *t* gives the values of all model species at *t*. The state vector is updated in discrete time using the transition tables in Fig. 5 for each species. At each update step, the molecular species are updated asynchronously in order to better reflect noisy biological processes. In the same vein, a species is updated with probability *p* < 1, since not all possible interactions are assumed to occur at each time step. Finally, each species is only allowed to jump a maximum of one level at any time step, allowing for a continuous transition between levels for the system during the course of the simulation. The system attains a steady state when the state vector remains constant during incremental time steps.

**Figure 6:**
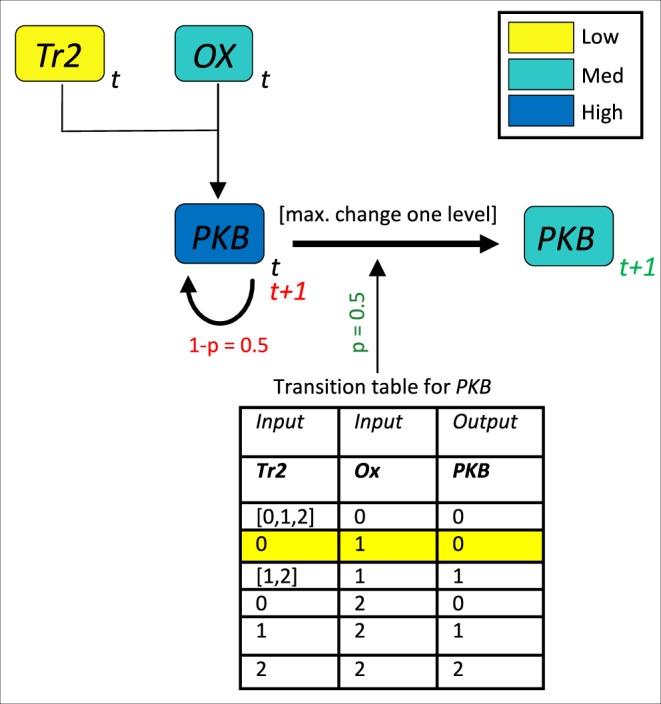
Example of an instantiation of the update function for *PKB*. The colors of the boxes indicate their activity level (high, medium, or low), and the time is indicated on the lower right-hand corner of each variable box. The value for *PKB* at time = *t* + 1 can either be medium (if the update rule is enacted, highlighted by the green coloring of the time and update probability), or low (if the update rule is not enacted, highlighted by the red coloring of time and probability).

We consider an expository example: the transition table for *PKB* (Fig. 5e) shows that *Tr*2 and *Ox* both contribute to *PKB* activation. Let us assume that *PKB* at the current time, *t*, is high, *Tr*2 is low, and *Ox* is medium (Fig. 6). According to the transition table, *PKB* should transition to low activity at the next time step. Due to the continuity constraint, *PKB* will drop to a medium level, but only if the update rule is enacted, since there is some probability, (1 − *p*), that *PKB* will not update and remain at its original high level.

We shall now discuss the derivation of each of the rules in the multistate rule-specification transition tables depicted in Fig. 5.

### The TRAF proteins

Both OX40 and 4-1BB bind and activate TRAF2^29,58–60^, while OX40 has also been shown to activate TRAF5 and TRAF3^29,58^, and 4-1BB, TRAF1^58^. It has been shown that TRAF3 acts to negatively regulate TRAF2-mediated downstream-signaling by competing with TRAF2 for binding with OX40^61^. To reduce the overall number of variables in the model, we do not model TRAF3 directly, but we make the effect of 4-1BB on TRAF2 activation to be stronger than that of OX40 to account for the added action of TRAF3 on OX40-mediated TRAF2 activation. Finally, as it has been found that TRAF1 is involved in the promotion of TRAF2 activity^62^, we incorporate this effect into the function for TRAF2. We construct the update rules for the species representing TRAF2 (*Tr*2) as follows. We first create a *stim*_*T*_ variable that combines the effects of 4-1BB (*I*) and OX40 (*Ox*), with *I* having the stronger effect on the value of *stim*_*T*_. We set the temporary value of *Tr*2 == *stim*_*T*_. Next, TRAF1 (*Tr*1) can boost (or, mitigate if it is = 0) *stim*_*T*_ to generate the final update value of *Tr*2 (Fig. 5b). The activity of *Tr*1 and TRAF5 (*Tr*5) are set to be proportional to *I* and *Ox* activity, respectively (Fig. 5a,c).

### NF-*κ*B

The NF-*κ*B pathway, which controls cell survival and inflammatory pathways, is activated by both OX40 and 4-1BB in a TRAF-dependent manner^58,59^. TRAF2-mediated activation of the canonical NF-*κ*B pathways has been well characterized^59,63^, but a dominant-negative mutant of TRAF5 has also been shown to reduce OX40-mediated NF-*κ*B activation^29^. Since the evidence for TRAF2-dependent activation of NF-*κ*B is stronger than for TRAF5, we model *Tr*2 to have a stronger effect on NF-*κ*B (*Nk*) activation than *Tr*5 (Fig. 5d).

### PKB, Bim, and JNK

Protein Kinase B (PKB) (also known as AKT) is a Serine and Threonine Kinase that is activated by the lipid kinase PI3K and is involved in the regulation of many central cellular processes, including cell survival^64^. It has been shown that OX40-mediated survival of activated CD4 T cells is dependent upon PKB activation^17^, which is dependent upon TRAF2 activity^65^. Nevertheless, 4-1BB-induced survival of CD8 T cells has been shown to be independent of the PI3k/PKB cascade^66^. While the former experiments were conducted in CD4 T cells, and the latter in CD8, until further evidence is presented, we assume that downstream signaling of activated OX40 and 4-1BB is mediated by the same molecular species. We thus model *PKB* activation as a process mediated by *Ox* and *Tr*2 (Fig. 5e).

The pro-survival effect of 4-1BB signaling is partially dependent on TRAF1 activity, which inhibits the pro-apoptotic BCL-2 family member Bim in an ERK-dependent pathway^67^. Additionally, in an ERK-independent pathway, PKB acts as an inhibitor of Bim via inactivation by phosphorylation of members of the FoxO transcription factor family, which promote Bim transcriptional upregulation^68,69^. The effect of Bim inhibitory inputs may be modulated by direct activation of Bim by c-Jun N-terminal Kinase (JNK) via phosphorylation, thereby leading to its release from dynein and myosin V motor complexes which sequester it from its pro-apoptotic activities^70^. JNK, a member of the mitogen-activated protein kinase (MAPK) family, is activated via a MAPK phosphorylation cascade^71^ that has been shown to be initiated by TRAF2 (via activation of higher level MAP4K AKT1, as well as other upstream MAPKs)^72–74^ (reviewed in^16^). NF-*κ*B has been shown to be an inhibitor of JNK activation in a manner that is independent of a downstream effect of caspase activation (which occurs upon NF-*κ*B inhibition) and is transcriptional in nature^75–78^. Hence, we take activity of *Bim* inhibitors (*inh*_*B*_) to be equal to the max(*Tr*1, *PKB*) and model *Bim* activation as a balance between *inh*_*B*_ and *JNK*, and *JNK* activity as a balance between activator *Tr*2 and inhibitor *Nk* (Fig. 5f,g).

### Survival and cytokine production

Apoptosis, or programmed cell death, can be initiated via several pathways that converge upon the activation of the caspase family of proteins, which enact the apoptotic process of cellular component degradation^79^. One pathway, termed the ‘intrinsic pathway’, occurs via Bcl2-family-mediated permeabilization of the mitochonrial outer membrane, which is a signal for initiation of the caspase cascade. The Bcl2 family contains both pro- and anti-apoptotic members, and it is the balance of these different species which determines where apoptosis is initiated or not via this pathway^80,81^. We have already discussed how 4-1BB signaling can lower the activity of Bim, a pro-apoptotic member of this family^30^. Activation of NF-*κ*B results in the enhanced expression of pro-survival members of the Bcl2 family, including A1/Bfl1 and Bcl-xL, as well as activation of other arms of the anti-apoptotic cellular machinery^16,82–84^. Additionally, apoptosis signaling pathways are activated by PKB^64^, and inhibited by JNK^85^ (independent of their respective action on Bim, discussed earlier). Therefore, survival is modeled as a balance between the levels of survival-promoting and inhibiting species (Fig. 5h). NF-*κ*B regulates production of cytokines by activated T cells, including IL-2, IL-6, IFN*γ*, and TNF*α*^84,86,87^. JNK has also been implicated in the production of IL-2 during T cell activation, but to differing extents depending on the study^88^. Therefore, we model cytokine production as a function of NF-*κ*B and JNK, with NF-*κ*B having the stronger positive effect (Fig. 5i).

## Supplementary information

Supplementary Information
