# Supplementary material for: A mathematical model of combined CD8 T cell costimulation by 4-1BB (CD137) and OX40 (CD134) receptors
Source: Sci Rep. 2019 Jul 26;9:10862. doi: 10.1038/s41598-019-47333-y (PMC6659676; doi:10.1038/s41598-019-47333-y)
Supplement: Supplementary file 1 — Supplementary Information [file 41598_2019_47333_MOESM1_ESM.pdf]

Supplementary Material

A mathematical model of combined CD8 T cell costimulation by 4-1BB (CD137) and OX40 (CD134) receptors

Anna Konstorum[1], Anthony T. Vella[2], Adam J. Adler[2], and Reinhard C. Laubenbacher[1,3]

[1]Center for Quantitative Medicine, School of Medicine UConn Health, 263 Farmington Ave., Farmington, CT

[2]Department of Immunology, School of Medicine, UConn Health, 263 Farmington Ave., Farmington, CT

[3]Jackson Laboratory for Genomic Medicine, 263 Farmington Ave., Farmington, CT

S1 Stochastic multistate discrete logic modeling framework: background

The mathematical model, which takes on a stochastic multistate discrete logic framework, consists of 9 variables (*Tr1*, *Tr2*, *Tr5*, *Nk*, *PKB*, *Bim*, *JNK*, *S*, and *C*) and two external inputs (*I* and *Ox*) that can take on values in $\{0,1,2\}$, which correspond to low (0), medium (1), and high (2) activity, respectively. External input values are set to a constant throughout the duration of the simulation, and the variable values are initialized as described below. The system at time t can be represented with the state vector

$$\mathbf{x}(t) = \{Tr1(t), Tr2(t), Tr5(t), Nk(t), PKB(t), Bim(t), JNK(t), S(t), C(t), I, Ox\},$$

where $x_i \in \{0,1,2\}$ for all $x_i \in \mathbf{x}$. Then, the state vector updates in discrete time as follows,

$$\mathbf{x}(t+1) = G(\mathbf{x}(t)) = \{g_i(\mathbf{x}(t)), p_i\}, \tag{S1}$$

where

$$g_i(\mathbf{x}(t)) = c(x_i(t), f_i(\mathbf{x}(t^*))). \tag{S2}$$

Each f_i is specified by the transition table for the variable x_i (Figure 5), c is a continuity constraint which limits the maximum jump size for each x_i to one level, and p_i indicates the probability P that $x_i(t+1)$ will update using g_i, i.e.

$$P(\mathbf{x}(t+1)) = \begin{cases} g_i(\mathbf{x}(t)) & \text{is } p_i \\ x_i(t) & \text{is } 1-p_i. \end{cases} \tag{S3}$$

We take $p_i = 0.5$ for all i. We use an asynchronous update scheme, so that each variable is updated in a random order at each time step, therefore $\mathbf{x}(t^*)$ represents the state of the system x during the random update scheme, whereby a subset of $x_i \in \mathbf{x}$ may have been updated. We note that the steady states for $G(\mathbf{x}(t))$ are equivalent to those for $G^*(\mathbf{x}(t)) = \{f_i\}$ since steady states do not depend on the asynchronous, stochastic, or continuous update scheme in this framework [1].

We consider again the expository example presented in the Methods: the transition table for *PKB* is found in Figure 5(e), and we replicate it in Table S1. We take $(Tr2, Ox, PKB)(t^*) = (0,1,2)$. Then, $f_{PKB} = 0$. Due to the continuity

constraint, $g_{PKB} = 1$. Then, $PKB(t+1) = 1$ if the system is updated (which it will be with probability $p_{PKB} = 0.5$).

Table S1: Transition table for *PKB*

Input	*Input*	*Output*
Tr2	*Ox*	*PKB*
$[0,1,2]$	0	0
0	1	0
$[1,2]$	1	1
0	2	0
1	2	1
2	2	2

S2 Simulation of the model

We used Matlab R2017A to simulate the system. Initial conditions for the simulations can be found in Table S2. We set initial TRAF activity to low (at time $t = 0$, $Tr1 = Tr2 = Tr5 = 0$) since our focus is on 4-1BB and OX40-mediated increases in TRAF activity, and hence the downstream effectors are also set to either low ($Nk = JNK = PKB = 0$) or to moderate ($Bim = 1$) since Bim is repressed by TRAF1. We note that randomizing the initial conditions has no effect on the results (1000 simulations with random initial conditions were run, with a resulting zero variance for the steady state of all variables).

Table S2: Initial conditions (I.C.s) for the dual costimulation model.

**Variable**	**I.C.**	**Variable**	**I.C.**	**Variable**	**I.C.**
TRAF proteins		Downstream effectors		Outcome measures	
Tr1	0	*Nk*	0	Survival (*S*)	0
Tr2	0	*Bim*	1	Cytokines (*C*)	0
Tr5	0	*JNK*	0		

S2.1 Simulating knock-out and overexpression experiments

For *ERK* knock-out, we change the update rule for *Bim* inhibitors to only include *PKB*: if *PKB* is at its highest level (2), then we set $Inh_B = 1$, else we set $Inh_B = 0$. In this way, we remove the action of *Tr1* towards *Bim* inhibition (since this inhibition is *ERK* dependent) and we do not allow maximal inhibition to occur even at high *PKB* levels since we assume that maximal *Bim* inhibition requires activation of *ERK* activity. For *ERK* overexpression, we set $Inh_B = 2$. In this way, we give a stronger weight to *ERK*-dependent mechanisms of *Bim* modulation over *ERK*-independent mechanisms. This is due to the presence of direct evidence that *ERK* is involved in 4-1BB (*I*)-mediated *Bim* inhibition [2]. We note that we do not model *ERK* directly as this would require us to distinguish between the strength of ERK-dependent and independent *Bim* inhibition under normal physiological conditions of *ERK*. For *PKB*, *JNK*, and *Nk* over-expression and inhibition, we set the respective variables either to 2 (overexpression) or 0 (inhibition) throughout the duration of the simulation.

References

[1] David Murrugarra, Alan Veliz-Cuba, Boris Aguilar, Seda Arat, and Reinhard Laubenbacher. Modeling stochasticity and variability in gene regulatory networks. *EURASIP J Bioinform Syst Biol*, 2012(1):5, Jun 2012.

[2] Laurent Sabbagh, Gayle Pulle, Yuanqing Liu, Erdyni N Tsitsikov, and Tania H Watts. ERK-dependent Bim modulation downstream of the 4-1BB-TRAF1 signaling axis is a critical mediator of CD8 T cell survival in vivo. *J Immunol*, 180(12):8093–101, Jun 2008.
